# Application of a Nomogram for Predicting the Risk of Subchorionic Hematoma in Early Pregnancy With In Vitro Fertilization-Embryo Transfer/Frozen Embryo Transfer

**DOI:** 10.3389/fendo.2021.631097

**Published:** 2021-03-02

**Authors:** Ma Yue, Linna Ma, Yurong Cao, Jun Zhai

**Affiliations:** ^1^Center for Reproductive Medicine, The First Affiliated Hospital of Zhengzhou University, Zhengzhou, China; ^2^Henan Key Laboratory of Reproduction and Genetics, The First Affiliated Hospital of Zhengzhou University, Zhengzhou, China; ^3^Henan Provincial Obstetrical and Gynecological Diseases (Reproductive Medicine) Clinical Research Center, The First Affiliated Hospital of Zhengzhou University, Zhengzhou, China

**Keywords:** subchorionic hematoma, IVF-ET, FET, nomogram, decision curve analysis

## Abstract

**Background:**

Subchorionic hematoma (SCH) is common in early pregnancy achieved by *in vitro* fertilization-embryo transfer/frozen embryo transfer (IVF-ET/FET), and is associated with adverse obstetric outcomes. However, there are no methods known to accurately predict the occurrence of SCH.

**Objective:**

To establish a nomogram prediction model for predicting the risk of SCH in early pregnancy with IVF-ET/FET and to analyze pregnancy outcomes of patients with SCH.

**Methods:**

Patients who underwent IVF-ET/FET treatment and were diagnosed with clinical pregnancy were enrolled in our study. A total of 256 patients with SCH were enrolled in the SCH group, and 526 patients without SCH in the control group. Logistic regression was used to screen risk factors for SCH, and the nomogram was developed according to the regression coefficient of relevant variables. Discrimination, effect, calibration, and the predictive model’s clinical usefulness were assessed using the C-index, the area under the receiver operating characteristic standard curve, calibration plot, and decision curve analysis. Internal validation was assessed using bootstrapping validation. The effects of SCH on pregnancy outcomes were analyzed.

**Results:**

A multivariate logistic regression analysis showed that fresh embryo transfer, polycystic ovary syndrome, hydrosalpinx, and thin endometrium were risk factors affecting the occurrence of SCH. Based on the above factors, a predictive model for the risk of SCH was created. The model displayed good discrimination, with a C-index of 0.783 (95% confidence interval: 0.750–0.816), area under the receiver operating characteristic standard curve of 0.783, and good calibration. A high C-index value of 0.765 could still be reached in the interval validation. Decision curve analysis showed that the nomogram was clinically useful when the intervention was decided at the SCH possibility threshold of 4%–87%. For patients with successful deliveries, the occurrence of SCH did not influence the gestational weeks of delivery, mode of delivery, preterm birth, height, and weight of the newborn.

**Conclusion:**

We screened the risk factors for SCH in patients who underwent IVF-ET/FET treatment. Successful establishment of a nomogram can effectively predict the occurrence of SCH. Furthermore, the incidence of miscarriage is higher in patients with SCH.

## Introduction

Subchorionic hematoma (SCH) in early pregnancy is a commonly observed feature on ultrasound. The mechanism causing SCH is believed to be the partial detachment of the chorionic membrane from the decidual membrane, resulting in blood accumulation between the chorionic membrane and the sacral membrane, resulting in the formation of a hematoma (1). Under pelvic ultrasound, SCH usually manifests as a hypoechoic or echo-free area between the chorionic membrane and the myometrium (mostly crescent-shaped). If the hematoma is large and clots have formed, petechial or linear hyperechoic areas can be detected around the gestational sac and under the chorionic membrane, whose lower edges are mostly connected to the inner cervical canal. The existence of SCH is possibly related to the occurrence of adverse obstetric outcomes, such as miscarriage (2). However, the induction of SCH remains unclear, and the occurrence of SCH is reportedly related to thrombosis (3).

Further studies have shown that SCH is associated with mesenchymal dysplasia and vaginal dysbacteriosis (4, 5). The reported incidence of SCH in pregnancy is 4%–48%, and clinical symptoms appear at 8–33 weeks of pregnancy (6). A study found that the incidence of SCH is higher in pregnancies achieved by *in vitro* fertilization and embryo transfer than in natural pregnancies (7).

Risk factors affecting SCH are unclear. There is a lack of intuitive and effective methods to assess the risk of SCH. The aim of the present study was to establish a nomogram for predicting the risk of occurrence of SCH in early pregnancies after *in vitro* fertilization-embryo transfer/frozen embryo transfer (IVF-ET/FET). The second objective was to evaluate the pregnancy outcomes of women with SCH.

## Materials and Methods

### Data Extraction

We retrieved data through the clinical reproductive medicine management system at the reproductive center of the First Affiliated Hospital of Zhengzhou University. During the study period from September 1, 2017, to October 31, 2017, 782 pregnancies achieved by IVF-ET/FET were included in the study. All patients underwent ultrasound examinations on days 35 and 45 after embryo transfer. Clinical pregnancy was defined as the presence of a gestational sac in the uterus by ultrasound. Under ultrasound, the SCH appeared as a crescent-shaped, sonolucent fluid collection between the chorion or placenta and the myometrium (8). Hydrosalpinx was defined as a distally occluded tube that was pathologically dilated or became pathologically dilated when patency was tested by ultrasound, hysterosalpingography, or laparoscopy (9). Preterm delivery was defined as delivery occurring at less than 37 weeks and more than 28 weeks of gestation, or birth weight ≥1,000g. Miscarriage was defined as delivery occurring at less than 28 weeks of gestation and birth weight ≤1,000g. A total of 256 patients with SCH were enrolled in the SCH group and 526 patients without SCH in the control group. Gonadotropin releasing hormone(GnRH) agonist down-regulation long protocols were used in the fresh cycle. The long-acting GnRH-a (33.75 mg, Triptorelin, Beaufour Ipsen, France) was used for pituitary downregulation on the 2^nd^ to 3^rd^ days of menstruation, and pelvic ultrasound and endocrine examination were re-examined after 28–35 days. If the endocrine indexes reached the standard for pituitary downregulation, 75–300 U of recombinant human FSH (Gonal-F, Merck Serono, Switzerland) were administered for controlled ovulation hyperstimulation. When the diameters of at least two dominant follicles were >18 mm or those of 2/3 follicles were >16 mm, 4,000–10,000 U of HCG was administered. At 36–38 h after injection, a transvaginal ultrasound-guided oocyte-pickup puncture procedure was performed. In the frozen-thawed embryo transfer, the natural cycle was used for patients with regular menstrual cycles. The hormone replacement cycle was used for patients with an irregular menstrual cycle or who were detected to have follicle non-growth or poor growth. Embryo transfer was performed on day 3 or day 5 after embryo formation. Luteal phase support was provided *via* vaginal administration of progesterone gel (Crinone, Merck Serono, Switzerland) once per day and oral dydrogesterone tablets (10 mg, Duphaston, Abbott, Netherlands) twice daily. Patients with recurrent spontaneous abortions, abnormal chromosomes, immune function abnormality, and a thrombosis history were excluded.

### Statistical Analysis

Statistical analysis was performed using SPSS 25.0 and R 4.0.1 software. Categorical variables were evaluated using a chi-square test, and continuous variables were assessed using the independent-samples Student’s t-test. We used logistic regression to screen the risk factors of SCH and the developed nomogram according to the regression coefficient of the relevant variables. The area under the receiver operating characteristic standard curve (AUC) was calculated to evaluate the prediction accuracy of the SCH nomogram model (10). Harrell’s C-index was evaluated to quantify the discrimination performance of the SCH nomogram (11), and bootstrapping validation was conducted to calculate a relatively corrected C-index (12). A calibration curve was used to measure the calibration of the SCH nomogram (13). Decision curve analysis was conducted to evaluate the clinical usefulness of the SCH nomogram by assessing the net benefits at different threshold probabilities (14). The types of investigated pregnancy outcomes included miscarriage rates (both early and late miscarriages), gestational weeks, neonatal weight, neonatal height, preterm birth rate, and mode of delivery. A *P* value <0.05 was considered statistically significant.

## Results

### Patients’ Characteristics

The baseline characteristics were similar between the two groups (**Table 1**). The prevalence of SCH in the study was 32.7% (256/782). Among the patients included in the study, 390 fresh and 392 frozen-thawed embryo transfers met the inclusion criteria. The frequency of SCH was higher with fresh embryo transfers (36.41% vs. 29.08%, p=0.029). The endometrium thickness before transfer (the day of HCG administration) was thinner in the SCH group (11.44 ± 2.86 vs. 11.95 ± 2.0, p=0.013). Of the women who had fresh embryo transfers, the number of ova obtained was higher in the SCH group (13.32 ± 6.56 vs. 11.99 ± 5.46, p=0.032). According to the infertility diagnosis, pregnancies in the study were divided into uterine malformation, uterine leiomyoma, intrauterine adhesion, hydrosalpinx (after the laparoscopic proximal tubal ligation), endometriosis, polycystic ovary syndrome (PCOS), male factor, and oviduct obstruction groups. The incidence of SCH was higher in the hydrosalpinx (40.24% vs. 30.74%, p=0.021) and PCOS (38.86% vs. 30.47%, p=0.026) groups than in the other groups (**Table 2**).

**Table 1:** Baseline characteristics.

Factor	SCH group (n = 256)	Control group (n = 526)	t value/χ^2^ value	P value
Age^a^(years)	30.29 ± 4.80	30.51 ± 4.74	0.613	0.540
BMI^a^(kg/m^2^)	28.23 ± 5.85	28.47 ± 5.56	0.571	0.568
AMH^a^(ng/ml)	3.99 ± 2.99	3.92 ± 3.24	0.869	0.771
FSH^a^(IU/L)	6.88 ± 2.40	6.71 ± 2.20	0.937	0.349
Infertility duration^a^ (years)	3.73 ± 2.51	3.71 ± 3.23	0.074	0.941
Infertility type^b^ (n,%)				
Primary Secondary	132(33.25)	265(66.75)	0.096	0.756
	124(32.21)	261(67.79)		
Type of embryo transfer^b^ (n, %)				
Fresh embryo transfer	142(36.41)	248(63.59)	4.768	0.029
Frozen-thawed embryo transfer	114(29.08)	278(70.92)		
Number of gestational sacs^b^ (n, %)				
Single gestational sac	173(34.60)	352(65.40)	0.034	0.854
Multiple gestational sac	83(28.26)	174(71.74)		
Duration of Gn^a^ (days)	13.83 ± 5.74	13.87 ± 5.73	0.312	0.967
Dose of Gn^a^ (IU)	2264.06 ± 1036.04	2431.35 ± 931.99	0.826	0.411
The number of ova obtained^a^	13.32 ± 6.56	11.99 ± 5.46	2.158	0.032
Oestradiol level^a^ (pg/mL)	3852.16 ± 1693.88	3745.95 ± 1727.17	0.590	0.556
Type of Frozen-thawed transfer^b^ (n, %)				
Natural cycle	45(27.43)	119(72.57)	0.369	0.544
Hormone replacement cycle	69(30.26)	159(69.74)		
Endometrium thickness^a^ (mm)	11.44 ± 2.86	11.95 ± 2.60	2.494	0.013

^a^Mean ± SD, comparison with the use of independent-samples T test.

^b^Using Chi-square test.

SCH, subchorionic hematoma; BMI, body mass index; AMH, anti-müllerian hormone; FSH, follicle stimulating hormone; Gn, Gonadotropin.

**Table 2:** Infertility diagnosis.

Diagnosis	SCH group (n, %)	Control group (n, %)	χ^2^ value	P value
Uterine malformation				
Yes	25(9.77)	58(11.03)	0.289	0.591
No	231(90.23)	468(88.97)		
Uterine leiomyoma				
Yes	54(21.09)	118(22.43)	0.180	0.671
No	202(78.91)	408(77.57)		
Intrauterine adhesion				
Yes	15(5.86)	31(5.89)	0.000	0.985
No	241(94.14)	495(94.11)		
Hydrosalpinx^a^				
Yes	66(25.78)	98(18.63)	5.312	0.021
No	190(74.22)	428(81.37)		
Endometriosis^a^				
Yes	65(25.40)	152(28.90)	1.056	0.304
No	191(74.60)	374(71.10)		
PCOS^a^				
Yes	82(32.03)	129(24.52)	4.925	0.026
No	174(67.97)	397(75.48)		
Male factor^a^				
Yes	63(24.61)	133(16.92)	0.042	0.838
No	193(75.39)	393(83.08)		
Oviduct obstruction^a^				
Yes	68(26.56)	137(26.05)	0.024	0.877
No	188(73.44)	389(73.95)		

^a^Using Chi-square test.

SCH, subchorionic hematoma; PCOS, polycystic ovary syndrome.

### Logistic Regression Analysis and Development of a Nomogram Prediction Model

**Figure 1:**
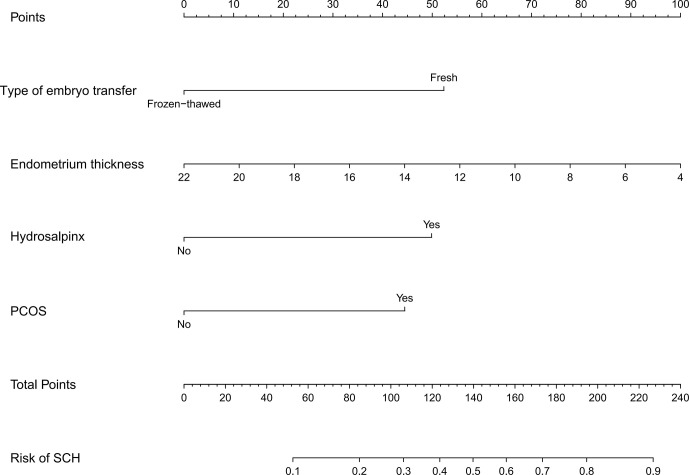
Developed SCH nomogram. The SCH nomogram was developed with type of embryo transfer, endometrium thickness, hydrosalpinx and PCOS. SCH, subchorionic hematoma; PCOS, polycystic ovary syndrome.

Logistic regression analysis demonstrated that fresh embryo transfer, hydrosalpinx, PCOS, and thin endometrium were independent risk factors for SCH (**Table 3**). The prediction model was developed based on these factors and presented as a nomogram (**Figure 1**).

**Table 3:** Multivariate logistic regression analysis.

Factor	Regression coefficients (β)	Significance (P)	OR	OR 95% confidence interval
Hydrosalpinx	0.369	0.044	1.45	1.01–2.07
PCOS	0.336	0.048	1.40	1.00–1.95
Cycle type	0.355	0.023	1.43	1.05–1.94
Endometrial thickness	0.075	0.011	0.93	0.88–0.98

PCOS, polycystic ovary syndrome; OR, odds ratio.

### Apparent Performance and Clinical Use of the SCH Nomogram

**Figure 2:**
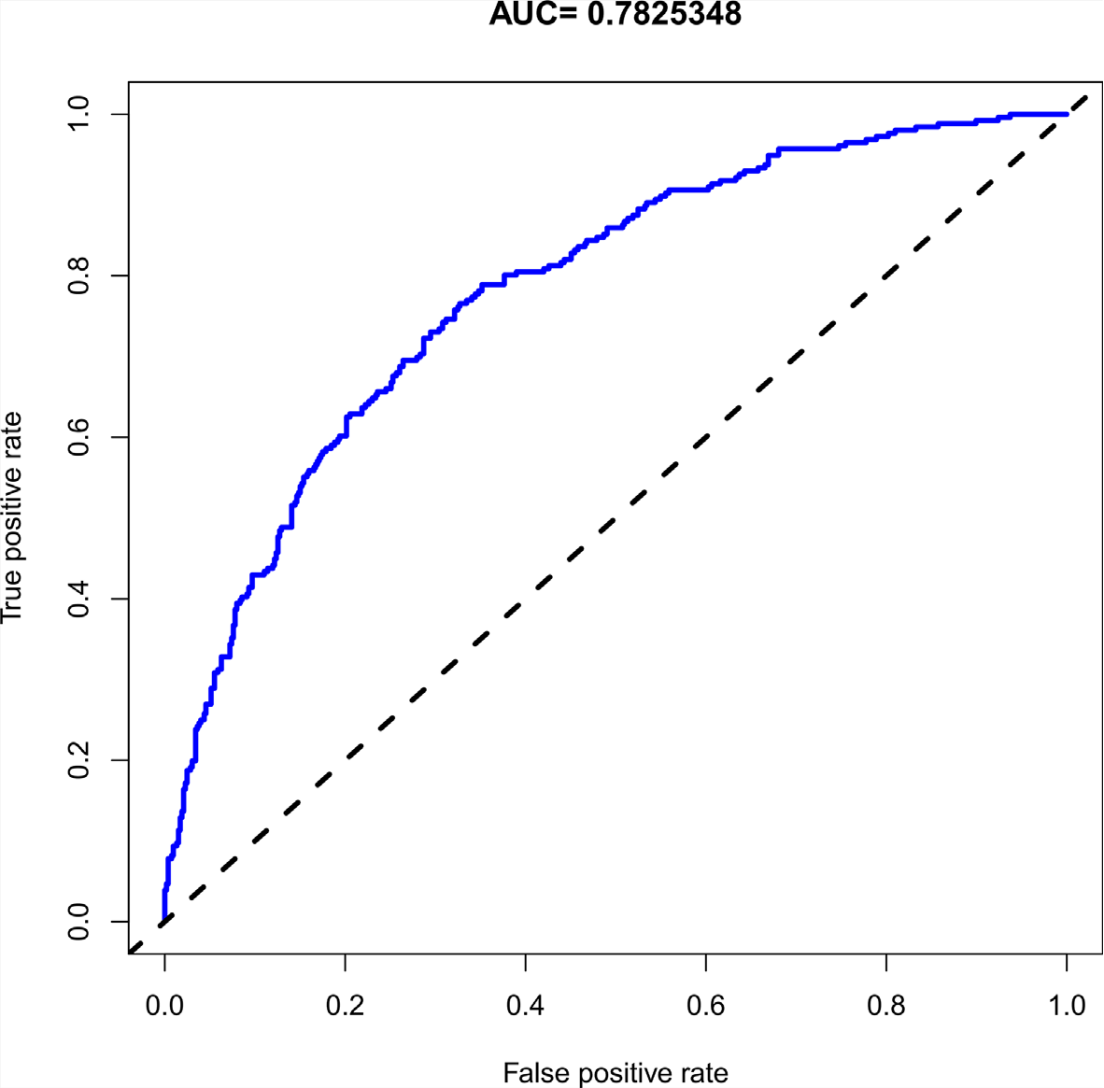
The receiver operating characteristic of the subchorionic hematoma (SCH) nomogram. AUC, the area under the receiver operating characteristic standard curve.

**Figure 3:**
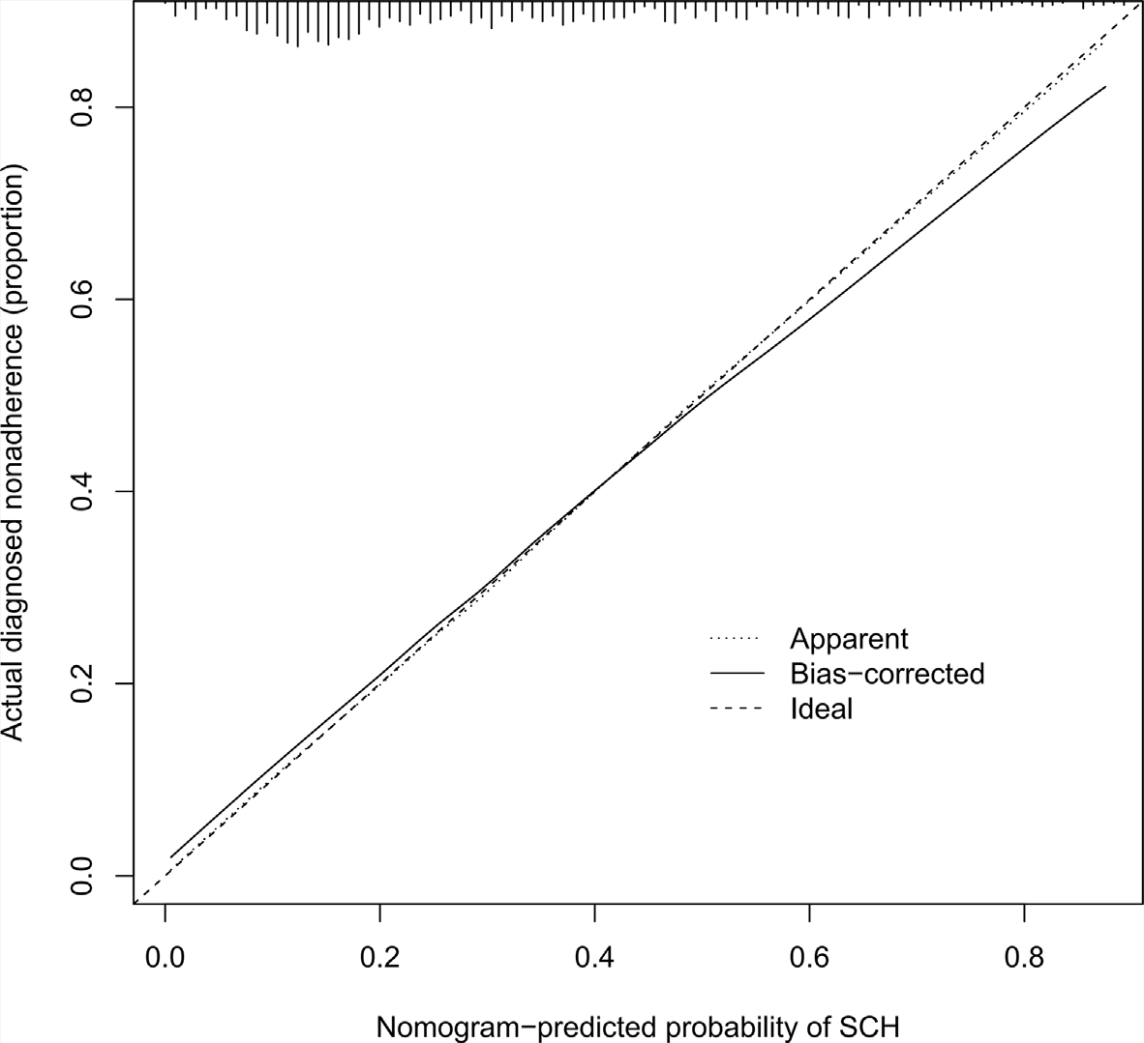
Calibration curves of the SCH nomogram. The x-axis represents the predicted risk for SCH. The y-axis represents the actual diagnosed SCH. The diagonal dotted line represents a perfect prediction by an ideal model. The solid line represents the performance of the nomogram, of which a closer fit to the diagonal dotted line represents a better prediction. SCH, subchorionic hematoma.

**Figure 4:**
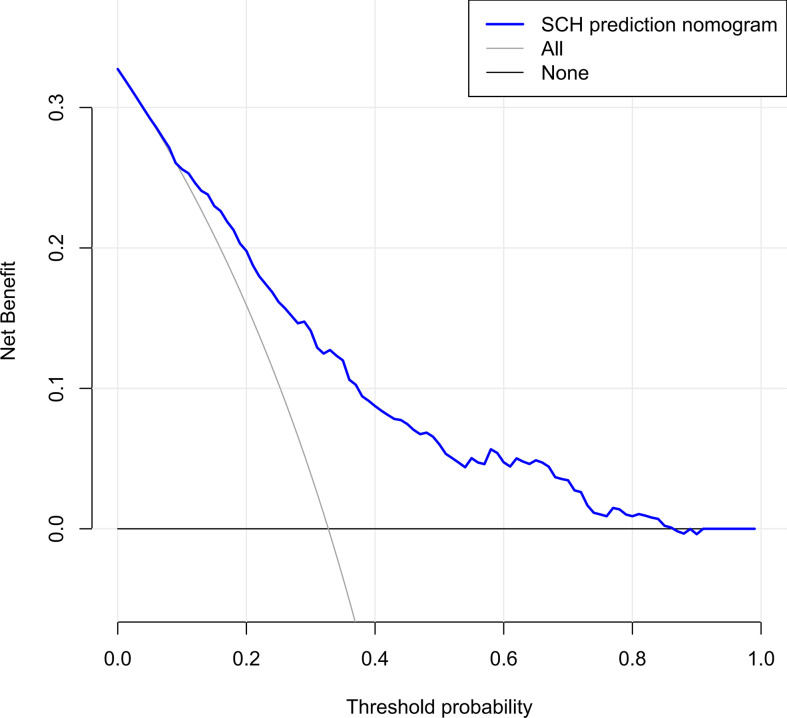
Decision curve analysis for the SCH nomogram. The y-axis measures the net benefit. The dotted line represents the SCH risk nomogram. The thin light solid line represents the assumption that all patients are diagnosed as SCH. The thin thick solid line represents the assumption that no patients are diagnosed as SCH. The decision curve showed that if the threshold probability of a patient and a doctor is ˃4 and <87%, respectively, using this nomogram in the current study to predict risk for SCH adds more benefit than the intervention-all-patients scheme or the intervention-none scheme. SCH, subchorionic hematoma.

The C-index for the nomogram was 0.783 (95% CI: 0.750–0.816) and was verified to be 0.765 through bootstrapping validation, which indicated that the model had great discrimination. The AUC of the nomogram was 0.783, suggesting a good prediction capability (**Figure 2**). The calibration curve of the nomogram for the prediction of SCH risk was proven to be in good agreement (**Figure 3**). The decision curve showed that if the threshold probability of a patient and a doctor is >4% and <87%, respectively, the use of the nomogram to predict SCH risk is more beneficial than the intervention-all-patient scheme or the intervention-none scheme (**Figure 4**).

### Pregnancy Outcomes

The miscarriage rate was significantly higher in the SCH group than in the control group (24.61% vs. 15.40%, P=0.002). However, the delivery gestational age, neonatal height, neonatal weight, preterm birth rate, and delivery mode were not significantly different between the two groups (the miscarriage pregnancies were excluded, **Table 4**). After excluding pregnancies with uterine malformation, PCOS, endometriosis, and hydrosalpinx that may influence the miscarriage rate, 219 patients with male factors and oviduct obstruction were retained. Among them, 63 pregnancies were enrolled in the SCH group and 156 were included in the control group. The miscarriage rate was still significantly higher in the SCH group(25.40% vs. 13.46%, P=0.035).

**Table 4:** Pregnancy outcomes.

Outcomes	SCH group (n = 256)	Control group (n = 526)	t value/χ^2^ value	P value
Miscarriage rates^b^ [%(n/N)]	24.61(63/256)	15.40(81/526)	9.723	0.002
Gestational weeks^b^ (week)	37.72 ± 2.17	37.94 ± 1.90	1.299	0.194
Neonatal weight^b^ (g)	3264.97 ± 474.60	3203.26 ± 494.59	1.476	0.140
Neonatal height^b^ (cm)	49.59 ± 1.99	49.88 ± 1.98	1.668	0.096
Preterm birth rate^b^ [%(n/N)]	16.58(32/193)	14.83(66/445)	0.317	0.574
Mode of delivery^b^ (n,%)				
Cesarean section	149(77.20)	355(79.78)	0.537	0.464
Natural delivery	44(22.80)	90(20.22)		

^a^Mean ± SD, comparison with the use of independent-samples T test.

^b^Using Chi-squared test.

SCH, subchorionic hematoma.

## Discussion

Subchorionic hematoma is common in pregnancies achieved by IVF-ET/FET; however, the mechanism causing SCH is unclear. Risk factors affecting the incidence of SCH and pregnancy outcomes of patients with SCH in assisted reproductive technology have remained unclear. Our study showed that the incidence of SCH was significantly higher in pregnancies achieved by fresh embryo transfer than in those achieved by frozen-thawed embryo transfers. PCOS, hydrosalpinx, and thin endometrium were independent risk factors affecting the occurrence of SCH, and the miscarriage rate increased significantly in patients with SCH.

Zhou et al. reported that the incidence of SCH was higher in patients receiving fresh embryo transfer (15). In contrast, Asato et al. showed that the incidence was higher in pregnancies achieved by frozen-thawed embryo transfer (7). However, most researchers believe that the incidence of obstetric complications is lower with frozen-thawed embryo transfer, and the obstetric outcomes are also better for frozen-thawed embryo transfers (16, 17). Some studies have suggested that the relatively natural womb environment during frozen-thawed embryo transfer is more conducive to embryo implantation and placenta formation (18, 19). In contrast, ovarian hyperstimulation in fresh embryo transfer affects the generation and implantation of endometrial blood vessels (20). A study showed that the incidence of vaginal bleeding in pregnancies achieved by fresh embryo transfer cycle increases with the number of ova obtained (21). This probably resulted from the higher estrogen levels in patients with more ova obtained, disturbing the function of endometrial blood vessels. Our study also showed that the incidence of SCH was higher in pregnancies achieved by fresh embryo transfer and increased with the number of ova obtained. The estradiol level was also higher in the SCH group, but the difference was not significant (p=0.556). As for the frozen-thawed embryo transfer cycle, the incidence of SCH was higher in the hormone replacement cycle, but the difference was not significant (30.26% vs. 27.43%, p=0.544). This could be due to the small sample sizes. Reich et al. also confirmed that the incidence was higher in the hormone replacement cycle (22). In addition, endometrium thickness on the day of HCG administration, which is correlated with endometrial receptivity, reflects the function of the endometrium and is associated with endometrial receptivity (23, 24). Our study showed that endometrium thickness was lower in the SCH group (p=0.013).

In the present study, all patients in the hydrosalpinx group were treated with tubal ligation in order to avoid the toxic effect and mechanical erosion of the hydrosalpinx fluid on the endometrium. Salpingectomy was not performed considering its potential impact on ovarian function. The hydrosalpinx could not return to the uterine cavity, resulting in toxic effects and mechanical erosion. Some pathogenic factors, such as cytokines, prostaglandins, leukocyte chemokines, and other inflammatory factors, are induced by the hydrops and act on adjacent organs through lymph and blood circulation, thus disrupting the function of the endometrium (25). The expression levels of integrin α_γ_β_3_, leukemia inhibitory factor, and homeobox gene A10 (HOXA10) are significantly decreased in patients with hydrosalpinx (26, 27). Therefore, hydrosalpinx might disturb endometrial receptivity in various ways, leading to SCH. Likewise, PCOS might induce SCH by influencing endometrial receptivity (28). Some studies have suggested that the expression of integrin α_γ_β_3_, interleukin 6 family cytokine (LIF), and HOXA10 genes decrease in patients with PCOS (29). Insulin resistance and hyperinsulinemia play a key role in PCOS physiology and pathology. Lathi et al. found that high insulin levels can reduce the expression of insulin-like growth factor-binding protein-1 (IGFBP-1) (30). A study showed that with high insulin levels, the expression of integrin and osteopontin also shows a decreasing trend (31).

Among studies on assisted reproduction in humans, few have evaluated the effects of SCH on pregnancy outcomes in women who underwent IVF-ET/FET (15, 32). A study reported that the presence of SCH in patients who underwent IVF-ET/FET had no influence on the incidence of spontaneous miscarriage or live birth rate (15). As for natural pregnancies, however, most studies have suggested a significant correlation between SCH and adverse obstetric outcomes (2, 33). Moreover, the occurrence of SCH could increase the risk of spontaneous miscarriage, premature delivery, placental abruption, and gestational hypertension (34–37). Based on our findings, SCH was also found to be an independent risk factor for miscarriage.

To the best of our knowledge, this was the first study that applied the nomogram prediction model in predicting the risk of SCH in early pregnancy with IVF-ET/FET. The nomogram had good predictive efficacy and guidance significance for clinical decisions. With the nomogram, we could intuitively assess the risk of SCH and administer individualized treatments for high-risk patients. For example, a patient with hydrosalpinx would have a score of 50 based on our nomogram, corresponding to a 10% risk of SCH. In a patient with a combination of two risk factors (PCOS and FET), the total score is 97, which corresponds to a 26% risk of SCH. Regarding patients with a high risk of SCH during the first trimester, monitoring should be enhanced to detect SCH as soon as possible and take timely measures to reduce the occurrence of miscarriage.

## Data Availability Statement

The raw data supporting the conclusions of this article will be made available by the authors, without undue reservation.

## Ethics Statement

Parameters such as medical history identifiers were recoded prior to extraction to maintain anonymized records, with no access to identifying information, in accordance with the principles of Good Clinical Practice and the Declaration of Helsinki. As such, the Ethics Committee for scientific research and clinical trials of the First Affiliated Hospital of Zhengzhou University approved the study.

## Author Contributions

MY, L-NM, and Y-RC performed the data collection, statistical analyses, and manuscript preparation. MY and JZ participated in the study design and manuscript preparation. All authors contributed to the article and approved the submitted version.

## Funding

Funding for the work undertaken in this study was provided by a Project Grant from the National Natural Science Foundation of China (grant no. 82071649). The funders had no role in the study design, data collection and analysis, nor decision to submit the article for publication.

## Conflict of Interest

The authors declare that the research was conducted in the absence of any commercial or financial relationships that could be construed as a potential conflict of interest.

## Abbreviations

IVF-ET/FET, *in vitro* fertilization-embryo transfer/frozen embryo transfer; PCOS, Polycystic ovary syndrome; SCH, Subchorionic hematoma.
